# Constructing and validating an occupational job strain index based on five Norwegian nationwide surveys of living conditions on work environment

**DOI:** 10.1186/s12889-022-14957-1

**Published:** 2023-01-07

**Authors:** Giang Huong Le, Åsmund Hermansen, Espen Dahl

**Affiliations:** Faculty of Social Sciences, Department of Social Work, Child Welfare and Social Policy, OsloMet - Oslo Metropolitan University, Oslo, Norway

**Keywords:** Job exposure matrix, Psychosocial job exposures, Job strain, Reliability, Validity

## Abstract

**Background:**

It has been claimed that Nordic register data are a “goldmine” for research. However, one limitation is the lack of information on working conditions. Job exposure matrices (JEMs) are one solution to this problem. Thus, the three aims of this study were (i) to investigate the reliability of an occupation-based psychosocial JEM, i.e., a Job Strain Index (job strain or JSI abbreviated), (ii) to examine the construct and criterion-related validity of this measure of job strain (iii) and assesses the concurrent and the predictive validity of an occupation-based Job Strain Index for use in analyses of Norwegian register data.

**Method:**

The study utilized five waves of the nationwide Norway Survey of Living Conditions in the Work Environment with a total sample of 43,977 individuals and register data with a total sample of 1,589,535 individuals. Job strain was composed of items belonging to the two dimensions of Karasek’s DC model, job demands and job control (1979). The reliability of the JSI and its dimensions and components were investigated by measuring the degree of agreement (Cohen’s kappa), sensitivity, specificity, and internal consistency (Cronbach’s alpha). Construct validity was assessed by confirmatory factor analysis, and criterion-related validity was measured by concurrent validity and predictive validity. The selected concurrent criteria were self-reported survey information on long-term sick absence, anxiety, depression, and sleeping difficulty. The predictive criteria were register information on receipt of disability benefits, mortality, and long-term sick leave.

**Results:**

Agreement between individual and occupation-based job strain and components was fair to poor. The sensitivity and specificity of occupation-based job strain and its components varied from acceptable to low. The consistency of the items comprising job demand and job control was clearly acceptable. Regarding concurrent validity, significant associations between (both individual and occupational) job strain, and long-term sick leave and sleeping difficulty were observed for both genders. Occupation-based job strain indicated an elevated risk for anxiety and depression among men, but not among women. As for predictive reliability, significant associations between occupation-based job strain and all three health outcomes were observed for both men and women.

**Conclusion:**

Our occupation-based JSI serves as a reliable and valid indicator of psychosocial job exposure that can be used in analyses of Norwegian register data where individual information on such conditions is missing.

## Introduction

In Nordic countries, national register data have been a valuable source of information for research for decades. They have even been dubbed “a goldmine” for research [1] as they include longitudinal data on the entire national populations, characterized by numerous variables and covering a wide range of life domains. However, one limitation is the lack of information on working conditions. To remedy this shortcoming, scholars have constructed job exposure matrices (JEMs) to create information on work environments, not for individuals, but for job titles [2]. With a history dating back to the 1980s, JEMs have proven useful in research on data where such information is missing [3, 4]. The JEM method is cost-effective; it provides systematic, unbiased, and reproducible results, and renders objective job-related information on exposures, in contrast to the subjective information given by respondents in surveys [3]. However, this approach is not without its challenges and pitfalls. One major problem with a JEM is that it entails the risk of misclassification, which may limit its applicability. This relates to the exact definition of exposures, as well as the classification of exposed or non-exposed. Job exposure matrices do not take into account the variation in working tasks and activities or differences in working locations over time or between workers with the same job titles [3, 5].

This raises questions about the reliability and validity of specific JEMs. This article investigated the statistical properties of an Occupational Job Strain Index (JSI) and its dimensions and components, based on Karasek’s Demand-Control Model [6]. The paper built on innovative work undertaken by Hanvold et al. [4], but exploited survey data with a much larger number of observations and, in addition, used register data. Hence, our study moved beyond Hanvold et al. by obtaining higher precision in the survey estimates, as well as benefitting from test results from a different and independent data source, register data. Coming from traditions linked to social policy, health inequality research and labour market analysis, with less focus on clinical effects of single exposures, or how specific exposures are associated with specific diagnoses (e.g. [4]), we have developed a broad Occupational Job Strain Index. The ultimate purpose behind the construction of the Occupational Job Strain Index is to create a measure which can be used in future analyses of Norwegian register data.

### Previous research

A substantial number of current studies have constructed and evaluated the reliability and validity of a psychosocial JEM. The reliability of the JEM was mainly reported by indicators, such as the internal consistency of the constructed JEM, kappa statistics to test the agreement between individual-based and occupational-based job exposure, and sensitivity and specificity to report the ability of constructed JEM to identify the exposure or non-exposure individuals, respectively. Psychosocial exposures at work are mostly described by the dimensions of Karasek’s models, including job demand and job control, which were commonly reported to have satisfactory internal consistency [7, 8]. A validation of alternative formulations of job strain supported using a continuous index when investigating health outcomes instead of the more common quadrant approach based on dichotomies, which inevitably will lead to loss of information [9]. The performance of the constructed psychosocial JEM varied across countries, which was reported as good for both job demand and job control in Australia [10], low for job control and bad for job demands in France [11], and good for job control and job strain in Finland [12]. The accuracy of detecting job exposure has been reported differently between genders [11, 12], but mostly suggests that the ability to identify psychosocial job exposure is better for women than for men. The reliability of the JEM was found to be different among exposures, which is likely to be higher for job control and job strain than for job demand [13].

The validity of the constructed JEM was tested by evaluating criterion-related validity using large population data. Based on solid evidence about possible links between psychosocial work exposures, especially high job strain (high job demand and low job control), and the risks of ill health, i.e., sickness absence [14], disability pension [15], and cardiovascular diseases [16] and different mental disorders [17, 18], such as risk for depressive symptoms [19, 20] and sleeping problems [21, 22], various health outcomes were taken into account to examine the reproducible likelihood of the constructed JEM compared with individual-based job exposure and the predictive validity of the JEM based on register data. The assessment of psychosocial work factors measured by JEM can also help to answer the question of whether the relationship between exposure and outcome is consistent regardless of the method used [16].

Recent Scandinavian studies have constructed and validated the JEM based on Karasek’s Demand-Control Model (1979), using large population data, such as the Danish JEM based on Work Environment Cohort Study data, including all patients aged 18–65 who received depressive and anxiety disorder treatments [23], the Swedish JEM using a large study population of all individuals aged 30–54 [24], the Finnish JEM utilizing the Health 2000 Study, and the Finnish National Work and Health Surveys [12]. The results showed the ability of a constructed JEM to predict various health outcomes, i.e., anxiety disorders [23], depression [12], sickness absence, and disability pension [24], with different patterns between men and women.

With respect to the context of Norway, a previous study by Hanvold et al. [4] utilized data of the work environment in 2006 and 2009 to construct group-based exposure estimations and to assess psychosocial JEM performance. The constructed JEM showed fair to poor agreement with the different performances between genders, reported to be higher among women than men [4]. The constructed JEM in Hanvold et al.’s study showed a good ability to identify occupations that are exposed to job strain, job control, and job demand. However, this study only investigated the concurrent validity of psychosocial occupational-level job exposure on low back pain.

This study used five waves of the Norwegian nationwide Survey of Living Conditions on work environment. This pooled dataset was used to examine four aspects of reliability (i.e., agreement, consistency, sensitivity, and specificity) associated with the JSI and its dimensions and components. Survey data were further used to assess the construct validity by means of factor analysis and the concurrent validity of the JSI, based on both individual based and occupational based exposures, using individually reported “long term sick leave”, “anxious symptoms”, “depressive symptoms” and “sleeping difficulty symptoms” as health outcomes. Finally, we assessed the predictive validity of the JSI for the entire working-age population in Norway, using register data and “disability benefits”, “mortality”, and “number of long-term sick absence periods” as health indicators. Where appropriate, the analyses were stratified by gender, as current research has shown divergent effects of work stressors on men and women [18].

## Methods

### Study population

This study utilized five surveys of the Norwegian nationwide Survey of Living Conditions on work environment from 2006, 2009, 2013, 2016, and 2019, with a total sample of 43,977 individuals. The purpose of using five surveys is to reach a larger number of observations, which may increase the accuracy of JEM performance. Data collection was conducted by Statistics Norway. The personal interviews conducted by telephone with computer assistance are on average 24–30 min long. Less than 0.5% of the interviews were conducted face-to-face. Since 2006, the survey on work environment has been funded by the Ministry of Labor and Social Inclusion to expand the sample and develop the survey as a panel.

The sample of the Norwegian nationwide Survey of Living Conditions on work environment was randomly drawn from the population aged 18–69 years, which represented active working-age people in the country. In the 2006 survey, the number of observations was 12,550 (with 67.2% response rate); in the 2009 survey, the number was 12,555 (with 61% response rate); in the 2013 survey, the number was 10,857 (with 53.1% response rate); in the 2016 survey, the number was 10,665 (with 52.6% response rate); and in the 2019 survey, the number of observations was 11,212 (with 57% response rate).

In 2007, the register data population consisted of people aged 18–55 who had a valid occupational code. In total, this included 1,589,535 individuals. Tables 1 and 2 below show the background characteristics of the study population. In both the survey data and register data, the number of men was slightly higher than women (23,062 men and 20,915 women in the survey data, 819,232 men and 770,303 women in the register data). The survey data had a lower proportion of respondents aged 25–44 but a higher proportion of respondents aged 45–69 (43.6% of the total sample aged 25–44 and 46.2% of the total sample aged 45–69) than that of the register data (56.2% and 29.2% of the total sample, respectively). The respondents in the survey data have a higher educational level than the population in the register data, as 42.5% of total respondents in the survey data have college or university education, compared with 34.1% in the register data. However, the distribution of the major occupational groups in both samples was not likely to be different.

**Table 1:** Background characteristics of the study population (survey data)

	All (*N* = 43,977)		Men (*N* = 23,062)		Women (*N* = 20,915)	
	*N*	%	*N*	%	*N*	%
**Age (years)**						
17–24	4,484	10,2	2,308	10,0	2,176	10,4
25–44	19,160	43,6	9,880	42,8	9,280	44,4
45–69	20,333	46,2	10,874	47,2	9,459	45,2
**Educational level**						
Primary school	11,116	25,3	5,979	25,9	5,137	24,6
Secondary/High school	14,007	31,9	8,524	37,0	5,483	26,2
College/university 4 years	13,328	30,3	5,508	24,9	7,820	37,4
College/university > 4 years	5,366	12,2	2,969	12,9	2,397	11,5
**Major occupational groups (STYRK-98)**						
Legislators, senior officials, and managers	4,569	10,4	3,032	13,1	1,537	7,4
Professionals	7,921	18,0	4,170	18,1	3,751	17,9
Technicians and associate professionals	11,818	26,9	5,236	22,7	6,582	31,5
Clerks	2,743	6,2	1,100	4,8	1,643	7,9
Service workers, shop and market sales workers	8,480	19,3	2,514	10,9	5,966	28,5
Skilled agricultural and fishery workers	822	1,9	670	2,9	152	0,7
Craft and related trade workers	3,911	8,9	3,665	15,9	246	1,2
Plant and machine operators and assemblers	2,552	5,8	2,235	9,7	317	1,5
Elementary occupations	1,161	2,6	440	1,9	721	3,5
**Long-term sick leave (previous month)**						
Yes	7,046	16,0	2,946	12,8	4,100	19,6
No	36,931	84,0	20,116	87,2	16,815	80,4
**Anxious symptoms**						
Severely/Somewhat	1,195	2,7	498	2,2	697	3,3
A little/Not at all	42,782	97,3	22,564	97,8	20,218	96,7
**Depressive symptom**						
Severely/Somewhat	1,021	2,3	430	1,9	591	2,8
A little/Not at all	42,956	97,7	22,632	98,1	20,324	97,2
**Sleeping difficulty symptoms**						
Severely/Somewhat	3,538	8,0	1,427	6,2	2,111	10,1
A little/Not at all	40,439	92,0	21,635	93,8	18,804	89,9

**Table 2:** Background characteristics of the study population (register data)

	All (*N* = 1,589,535)		Men (*N* = 819,232)		Women (*N* = 770,303)	
	*N*	%	*N*	%	*N*	%
**Age (years)**						
18–24	221,568	13,9	113,520	13,9	108,048	14,0
25–44	903,754	56,9	472,831	57,7	430,923	55,9
45–55	464,213	29,2	232,881	28,4	231,332	30,0
**Educational level**						
Primary school	321,207	20,2	176,392	21,5	144,815	18,8
Secondary/High school	714,616	45,0	399,202	48,7	315,414	41,0
College/university 4 years	424,436	26,7	167,405	20,4	257,031	33,4
College/university > 4 years	117,827	7,4	70,469	8,6	47,358	6,2
**Major occupational groups (STYRK-98)**						
Legislator, senior officials, and mangers	174,674	11,0	93,566	11,4	81,108	10,5
Professionals	188,963	12,0	101,577	12,4	87,386	11,3
Technicians and associate professionals	326,718	20,6	147,123	18,0	179,595	23,3
Clerks	125,183	7,9	50,160	6,1	75,023	9,7
Service workers, shop, and market sales workers	383,242	24,1	111,858	13,6	271,384	35,2
Skilled agricultural and fishery workers	9,810	0,6	7,176	0,9	2,634	0,3
Craft and related trade workers	170,450	10,7	161,664	19,7	8,786	1,1
Plant and machine operators and assemblers	127,104	8,0	107,531	13,1	19,573	2,5
Elementary occupations	83,391	5,24	38,577	4,7	44,814	5,8
**Disability benefits (2008–2017)**						
Yes	4,878	0,3	1,939	0,2	2,939	0,4
No	1,584,657	99,7	817,293	99,8	767,364	99,6
**Mortality (2008–2017)**						
Dead	18,467	1,2	11,484	1,4	6,983	0,9
Not dead	157,068	98,8	807,748	98,6	763,320	99,1
**Ten long-term sick leave periods or more (2008–2015)**						
Yes	428,510	26,9	152,019	18,6	276,491	35,9
No	1,161,025	73,1	668,213	81,4	493,812	64,1

As shown in Table 1, 16% of respondents in our survey data had experienced long-term sick leave during the previous 12 months. The percentage of respondents who experienced different mental health symptoms was 2.7% for anxiety, 2.3% for depression, and 8.0% for sleeping difficulty. More women than men in our survey sample reported different mental health problems.

As presented in Table 2, there is a low percentage receiving disability benefits, and mortality is low, with 0.3% and 1.2% of the study population, respectively. Approximately 27% of our register study sample took ten long-term sick leave periods or more during 2008 and 2015, 35.9% of women compared to 18.6% of men.

#### Constructing the job exposure matrix

In line with the previous study of Hanvold et al. [4], we constructed a gender-specific matrix with group-based exposure estimates at each intersection between occupations (rows) and psychosocial job exposures (columns) [4]. Hanvold et al. decided to have at least 19 respondents with the same occupational codes when constructing the JEM groups to enhance reliable estimates [4]. They reported that two of the authors grouped the occupations and discussed them further with a third author and two experts at the Norwegian Institute of Occupational Health. In total, they constructed 268 JEM groups based on occupational codes and answers from 18,939 respondents in the 2006 and 2009 surveys. Although this study used the same approach as Hanvold et al. to construct the JEM, we included a higher number of respondents, given the fact that we also included the 2013, 2016, and 2019 surveys. As a result, our study had a higher mean number of respondents in each JEM group, ranging from 176, as reported in Hanvold et al.’s study, to 412 in our study (Table 3). This table also shows a higher number of occupational codes (333 occupational codes) and a higher number of occupational codes with at least ⩾19 respondents (243 occupational codes). From 333 titles, we constructed the 268 JEM groups following Hanvold et al. [4].

**Table 3:** Number of occupational titles according to number of respondents and number of respondents per JEM group

	All (*N* = 333 (all)		Men (*N* = 317)		Women (*N* = 281)	
**Number of occupational titles according to number of respondents**	*N*	%	*N*	%	*N*	%
1–18	90	27	126	40	151	54
⩾ 19	243	73	191	60	130	46
Mean respondents per occupational title	132		73		74	
Min–Max respondents per occupational title	1	2224	1	831	1	1503
**Respondents per JEM group**	All (*N* = 268)		Men (*N* = 209)		Women (*N* = 195)	
Median	261		218		385	
Mean	412		276		562	
Min–Max	19	1,503	19	831	19	1,503

The construction of the 268 JEM groups was based on the occupational codes provided in our survey data. The Norwegian occupational standard is based on international classifications and follows the updated version of the international standard of the International Labor Organization. Data on occupations in the 2006 and 2009 surveys consist of 4-digit STYRK-98 codes, which are based on the International Standard Classification of Occupations, ISCO 88 [25]. In 2008, a new version of the International Standard Classification of Occupations 2008 (ISCO-08) was launched. Thus, Norway published a new Norwegian standard for occupational classification named STYRK-08, which is based on ISCO-08, with some adjustments in order to make the occupational classification suitable for occupations in the Norwegian labor market. This change led to differences in occupational codes between the previous surveys in 2006 and 2009 and the three later surveys in 2013, 2016, and 2019 [26].

Since our register data included the 4-digit STYRK-98 codes, we chose to transfer the 4-digit STYRK-08 to STYRK-98. There is no official table of correspondence between the 4-digit STYRK-98 codes and 4-digit STYRK-08 codes. When faced with the choice of having more than one STYRK-98 code to select, we chose to convert to the STYRK-98 code with the highest *N* in the 2006 and 2009 surveys combined. This applied to 28% of the 4-digit STYRK-08 occupational codes; thus, 72% remained unchanged.

### Variables

#### Constructing the job strain index

The JSI in our study is based on self-reported information with measured items for psychosocial exposures developed by the Statistics Norway (SSB). Following Karasek’s Demand-Control Model [6], the index is a combination of the psychological demand index (job demand) and decision-latitude index (job control). The measurement of psychological demands and job control followed the guidance of the General Nordic Questionnaire (QPS_Nordic_) [27]. In our study, psychological job demand was measured by four items: (1) quantitative demands, (2) conflicting ways of doing things, (3) insufficient resources, and (4) contradictory requests. Job control or decision-latitude was measured by six items: (1) decide how to go about the work, (2) decide the pace of work, (3) make important decisions, (4) use skills, (5) develop skills, and (6) monotonous work. The item variables were dichotomized as non-exposed and exposed, as described in Tables 4 and 5. Although the construction of Job Strain Index in our study is based on the idea of demand/control model by Karasek (1979), our measured items for psychosocial work exposure included only 10 items represented for two dimensions job demand and job control, compared to the original version of Job Content Questionnaire (JCQ) by Karasek (1979), which included 49 items to reflect the psychological job demands, job control, social support and other factors such as job insecurity, physical demands [28]. The measured items we used to construct the Job Strain Index in this study is thus a shortened version of JCQ, which is closer to the Swedish version [29]. The measured items for job strain in Swedish version are validated in the study of Chungkham et al. (2013) [30].

**Table 4:** Exposures, Questions, and Non-exposed or Exposed for Job Demand

Exposures	Questions	Non-exposed/Exposed
Quantitative demands	*How often do you have to skip lunch due to a heavy workload?* “Daily”, “a few days a week”, “once a week”, “a few days a month”, “never”	1 = Exposed (Daily, a few days a week, once a week), 0 = Non-exposed (A few days a month, never)
Conflicting ways of doing things	*How often do you have to do things you think should have been done differently?* “Very often or always”; “Quite often”, “occasionally”; “Quite rare”; “Very rarely or never”	1 = Exposed (Very often or always, quite often, occasionally), 0 = Non-exposed (Very rarely or never, quite rare)
Insufficient resources	*How often do you get job tasks without sufficient resources?* “Very often or always”; “Quite often”; “Occasionally”; “Quite rare”; “Very rarely or never”	1 = Exposed (Very often or always, quite often, occasionally), 0 = Non-exposed (Very rarely or never, quite rare)
Contradictory requests	*How often do you get contradictory requests from two or more people?* “Very often or always”; “Quite often”; “Occasionally”; “Quite rare”; “Very rarely or never”	1 = Exposed (Very often or always, quite often, occasionally), 0 = Non-exposed (Very rarely or never, quite rare)

**Table 5:** Exposures, Questions and Non-exposed or Exposed for Job Control

**Exposures**	**Questions**	**Non-exposed/Exposed**
Decide how to go about the work	*Can you decide yourself how to go about doing your work?* “To a very high degree”; “To a high degree”; “To some degree”; “To little degree”; “To very little degree”	0 = Non-exposed (To a high degree or to a very high degree), 1 = Exposed (To some degree, to little degree, to very little degree)
Decide pace of work	*To what extent can you decide your own work pace?* “To a very high degree”; “To a high degree”; “To some degree”; “To little degree”; “To very little degree”	0 = Non-exposed (To a high degree or to a very high degree), 1 = Exposed (To some degree, to little degree, to very little degree)
Important decisions	*Can you influence decisions that are important to your work?* “To a very high degree”; “To a high degree”; “To some degree”; “To little degree”; “To very little degree”	0 = Non-exposed (To a high degree or to a very high degree), 1 = Exposed (To some degree, to little degree, to very little degree)
Use skills	*What are the opportunities in your job to utilize the skills, knowledge and experience you have gained through education and work?* “Very good”; “Good”; “Bad”; “Very bad”	0 = Non-exposed (Very good, good), 1 = Exposed (Very bad, bad)
Develop skills	*How are the opportunities in your job to further develop skills in the areas you desire?* “Very good”; “Good”; “Bad”; “Very bad”	0 = Non-exposed (Very good, good), 1 = Exposed (Very bad, bad)
Monotonous work	*Does your work consist of constantly repeated work tasks?* “Almost all the time”, “About three quarters”; “Half the time”; “A quarter of the time”; “Never”	0 = Non-exposed (A quarter of the time, never), 1 = Exposed (Almost all of the time, about three-quarters, half the time)

Each item was dichotomized following the same procedure as Hanvold et al. [4], splitting each scale at the median to identify those who are exposed vs. non-exposed (see Table 4 and 5). Hanvold et al. underscores that defining those who are exposed, in the sense that the level of demands and control poses a health risk, is difficult. Thus, they decided to use the median as a cut-off, following Solovieva [12] which used the same approach in a Finnish validation study of a job exposure matrix for psychosocial factors. For the individual exposures, we calculated the median value for each item using the raw values and then used the median as a cut-off as to identify the exposed versus non-exposed individuals based on the individual information. The response categories defining exposed vs. non-exposed, which are shown in Table 4 and 5, are based on the median. In example for “Quantitative demands” the median value on the five-point scale was 2. Thus, those with a value above 2 (Daily = 5, a few days a week = 4, once a week = 3) were defined as exposed. Whereas for the occupation-based exposures, we calculated the share of exposed individuals for each item within each JEM group and used the median as a cut-off as to identify individuals defined as exposed and non-exposed based on their occupational code.

We constructed the psychosocial exposure variables in such a way that all variables reflected the proportion of individuals within each of the JEM groups being exposed. The scale of psychosocial exposure variables goes from 0–100%. The occupational codes with a value of 0 indicate that none of these occupational codes have provided an answer that involves exposure. The occupational codes with a value of 100 indicate that all respondents in this occupational code have provided an answer that involves exposure.

In the scholarly literature, job strain has been measured in numerous ways, the most common being the quadrant approach. However, a validation of alternative formulations of job strain shows that using a continuous variable measuring the degree of strain best predicts stress and back pain [9]. In accordance with this study and the fact that we do not want to lose information by dichotomizing continuous measures, as is the case with the quadrant approach, we constructed a continuous JSI. For the occupational based JSI, we first calculated the mean proportion of individuals within each JEM group reporting to be exposed on the four items measuring demands (see Table 4). A higher value represents a larger share within a JEM group reporting to be exposed to a high degree of demands. Secondly, we calculated the mean proportion of individuals within each JEM group reporting to be exposed on the six items measuring control (see Table 5). A higher value represents a larger share within a JEM group reporting to be exposed to lower degree of control. Thirdly, we added these two numbers together and divided by two. Accordingly, higher values on the index represent higher degrees of demand and lower degrees of control, whereas lower values represent lower degrees of demand and higher degrees of control. The individual JSI was calculated in the same manner, however using the individual based exposures.

#### Health outcome variables

To test the criterion-related validity of the psychosocial JEM, we examined the association between the JSI and different health outcomes based on both the survey and register data. Information on long-term sick leave and three different mental health symptoms, including anxiety, depression, and sleeping difficulty, were derived from survey data to test the concurrent validity of the constructed JSI. To ascertain the information on sick leave, the following question was asked: ‘During the last 12 months, have you had continuous sick leave of more than 14 days?’ ‘1. Yes, 2. No’. The anxious symptom was tapped by the question: “During the last month, have you been bothered by nervousness, anxiety, or restlessness?” “1. Very bad, 2. Pretty bad, 3. A little, 4. No”. The depressive symptoms were asked by question: “During the last month, have you been bothered by depression?” “1. Very bad, 2. Pretty bad, 3. A little, 4. No”. We recoded these two variables in such a way that people who answered, ‘very bad’ and ‘pretty bad’ were ‘exposed’, and people who answered, ‘A little’ and ‘No’ were ‘non-exposed’.

The sleeping difficulty symptom was asked by the question: “During the last three months, have you had difficulty sleeping because thoughts of work kept you awake?” “1. A few days a week, 2. About once a week, 3. A few times a month, 4. Seldom or never”. We recoded this variable such that people with sleeping difficulty symptoms ‘a few days a week’ and ‘about once a week’ were ‘exposed’ and those who experienced symptoms ‘A few times a month’ and ‘seldom and never’ were ‘non-exposed’. Information on long-term sick leave, mortality, and disability was obtained from register-based data to test the predictive validity of the occupational-level JEM. The long-term sick leave variable identifies individuals having ten long-term sick leave periods or more during 2008 to 2015. Disability was measured by whether individuals received disability benefits during the period 2008 to 2017. The mortality variable provided information on whether the individual died during the period 2008 to 2017.

### Results

#### Reliability of the occupation-based JSI

The reliability of the occupation-based JSI was compared with the individual-based JSI by three measures: Cohen’s kappa, sensitivity, and specificity. In addition, we assessed the internal consistency of the two dimensions of occupation-based job strain, job demands, and job control by means of Cronbach’s alpha. Cohen’s kappa was used to measure inter-rater reliability, or the agreement, between the individual exposures and occupation-based exposures. The kappa value could be interpreted as no agreement (≤ 0), poor (0.01–0.20), fair (0.21–0.40), moderate (0.41–0.60), good (0.61–0.80), and excellent (0.81–1.00) [31]. Cronbach’s alpha values > 0.70 are considered acceptable.

As shown in Table 6, Cohen’s kappa reported a ‘fair’ agreement between individual exposure and occupation-based exposure for job demand for women and ‘poor’ for men. For each exposure, the agreement scores were reported as ‘fair’ for quantitative demand for both genders (0.24 for men and 0.29 for women), but ‘poor’ for a conflicting way of doing things, insufficient resources, and contradictory requests. The Kappa statistics reported ‘fair’ for job control for both men and women (0.25 for men and 0.24 for women). For each exposure in job control, ‘fair’ agreement scores were applied to decide pace of the work (0.22 for both men and women), important decisions (0.23 for men and 0.20 for women), monotonous work (0.27 for men and 0.32 for women), and decide how to go about the work for only men (0.22). The agreement between the individual-based and the occupation-based job strain was ‘poor’ for both genders (0.19 for men and 0.16 for women). Sensitivity and specificity, respectively, measure the ability to detect exposed and non-exposed individuals. Using the median value, as a cut-off for both the individual based and the occupation-based exposures, gave a sensitivity of > 60% for all exposures for women, and 8 over 13 exposures > 50% for men. Our constructed JEM had a better ability to identify the exposure for job demand, job control, and job strain for women (sensitivity scores > 70%) than for men (sensitivity scores < 50%), while the ability to detect non-exposure for job demand, job control, and job strain for men (specificity scores > 70%) was higher than for women (specificity scores < 60%).

**Table 6:** Comparing occupation-based and individual-based psychosocial exposures. Cohen’s kappa, sensitivity and specificity measures, survey data

		Men			Women		
Exposures	**Cut-off**	**Kappa**	**Sensitivity**	**Specificity**	**Kappa**	**Sensitivity**	**Specificity**
Job Demand	Median	0.18	47	71	0.29	73	56
Quantitative demands	Median	0.24	69	56	0.29	64	66
Conflicting way of doing things	Median	0.12	52	60	0.15	65	50
Insufficient resource	Median	0.13	56	60	0.18	72	50
Contradictory requests	Median	0.10	60	54	0.12	63	54
Job Control	Median	0.25	48	76	0.24	78	45
Decide how to go about the work	Median	0.22	50	73	0.18	80	40
Decide pace of work	Median	0.22	48	73	0.22	78	43
Important decisions	Median	0.23	41	81	0.20	84	36
Use skills	Median	0.08	81	55	0.06	86	42
Develop skills	Median	0.15	64	64	0.10	81	41
Monotonous work	Median	0.27	68	61	0.32	70	63
Job Strain	Median	0.19	44	75	0.16	84	35

The internal consistency of the items that made up the occupation-based job demand dimension, measured by Cronbach’s alpha, was 0.73. For the occupation-based job control dimension, the alpha value was 0.85. This means that the internal consistency of both dimensions of occupation-based psychosocial exposure was acceptable.

#### The construct validity of the occupation-based JSI: confirmatory factor analysis

We performed a confirmatory factor analysis (CFA) to assess the construct validity of the two occupation-based psychosocial dimensions, job demand and job control. Given that numerous studies [7, 32, 33] have documented that job strain consists of the relation between two distinct and separate dimensions, we chose to perform a CFA for each dimension. Since potential gender differences were accounted for in the creation of the occupation-based job demands index and the job control index, Tables 7 and 8 include both men and women.

**Table 7:** Confirmatory factor analysis of occupation-based job demand (one-factor model)

	X^2^	P	RMSEA	CFI	TLI	SRMR	Correlated error
Original	1.481	0.477	0.000	1.000	1.004	0.011	
**Exposures**	*Standardized factor loading				**Standard error**		
**Share exposed—Quantitative demands**	.466				.049		
**Share exposed—Conflicting ways of doing things**	.784				.031		
**Share exposed—Insufficient resources**	.826				.029		
**Share exposed—Contradictory requests**	.713				.035		

^*^no cross-loadings and no correlated residuals

**Table 8:** Confirmatory factor analysis of occupation-based job control (one-factor model)

	X^2^	P	RMSEA	CFI	TLI	SRMR	Correlated error
Original	439.87	0.000	0.386	0.709	0.514	0.156	
**Important decisions** with **Develop skills** **Decide pace of work** with **Use skills** **Decide pace of work** with **Monotonous work** **Decide how to go about the work** with **Develop skills** **Decide how to go about the work** and **Develop skills** **Develop skills** with **Use skills** **Develop skills** with **Monotonous work** **Use skills** and **Monotonous work**	3.73	0.155	0.052	0.999	0.991	0.006	.536 -.360 -.355 .396 .772 .569 .563
**Exposures**	*Standardized factor loading				**Standard error**		
**Share exposed—Decide how to go about the work**	.911				.013		
**Share exposed—Decide pace of work**	.769				.059		
**Share exposed—Important decisions**	.928				.012		
**Share exposed—Use skills**	.589				.039		
**Share exposed—Develop skills**	.642				.035		
**Share exposed—Monotonous work**	.560				.040		

^*^no cross-loadings and no correlated residuals

The model evaluation was based on chi-square tests for model fit and further model fit indices, including the root mean square error of approximation (RMSEA), the comparative fit index (CFI), the Tucker-Lewis index (TLI), and the standardized root mean square residual (SRMR). For the model fit to be interpreted as acceptable, an RMSEA of < 0.05 was considered a close fit, while an RMSEA and an SRMR of up to 0.08 were considered acceptable. Comparing the fit of a target model with the fit of an independent or null model, the CFI had a cut-off for a good fit of ⩾ 0.90. A TLI of 0.95 indicates the model of interest and improves the fit by 95% relative to the null model, and the cut-off for good fit was TLI ⩾ 0.95. Furthermore, the correlations of residuals to improve the model fit were considered [34, 35]. Potential model adjustments were based on modification indices, as provided in the Stata output, using the ‘estat gof, stats (all)’ command. To obtain a clearer idea of the data and potential problematic items, a one-factor model was fitted to the data for both indices. To test whether modifications, in terms of correlated within-factor residuals, led to significant model improvement, modification indices were obtained using the ‘estat mindices’ command in Stata.

The results of fitting a one-factor model for the psychological demand index are shown in Table 7. The “Original” row shows the results when fitting the index with no cross-loadings and no correlated residuals. All factor loadings were moderate to high (i.e., > 0.4; see column “Standardized factor loading” in Table 7.) No modifications were needed to improve the model.

The results from fitting a one-factor model for the decision-latitude index are shown in Table 8. The “Original” row shows the results when fitting the index with no cross-loadings and no correlated residuals. All factor loadings were moderate to high (i.e., > 0.4; see column “Standardized factor loading” in Table 8). As shown, a model fit with eight modifications provides a satisfying model fit.

#### Concurrent validity of the JSI: survey data results

**Fig. 1:**
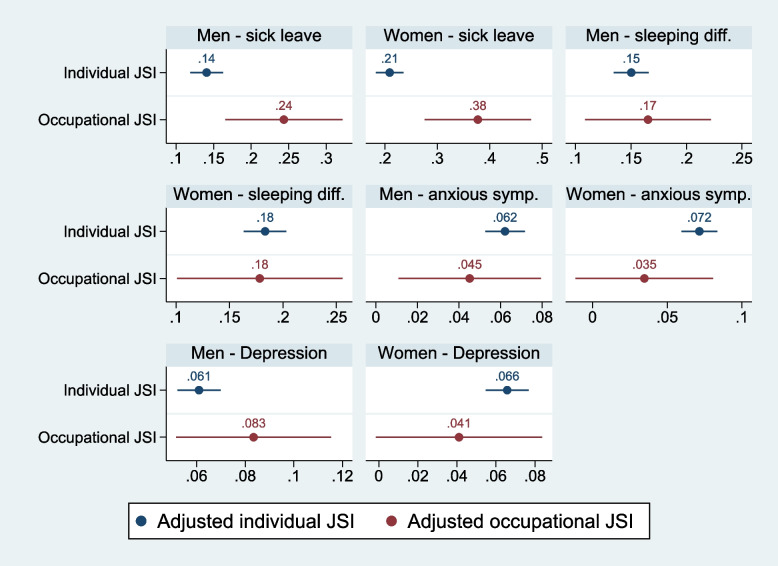
Linear probability model^*^ of individually reported long-term sick leave, sleeping difficulty, anxiety, and depression as dependent variables. Survey data. ^*^Results adjusted for level of education and age. Men *N* = 23,062, Women *N* = 20,915

Figure 1 presents the association between individual- and occupation-based JSI and self-reported sick leave, sleeping difficulty, anxiety, and depression. The models were gender-specific, with adjustments for educational level and age.

Regarding the concurrent validity of the JSI on long-term sick leave and sleeping difficulty, we found a reproducible likelihood for both men and women, as both the individual and the occupational-based JSI reported significant associations for both genders, and the occupational-level JSI estimates were not significantly different from the individual-based JSI estimates. As seen in Fig. 1, both the individual- and occupational-based JSI are significantly associated with anxiety and depression for men. As for women, the significant associations between job strain and anxiety, and job strain and depression were observed only for the individual-based JSI, but not for the occupation-based JSI. This means that the reproducible likelihood of anxious and depressive symptoms was reported only for men but not for women. Furthermore, our study also reinforces the current finding reported by a Danish study (Wieclaw et al., 2008) that the relation between psychosocial work exposures and depression may differ between genders. Thus, our study shows that the impact of psychosocial work exposures on mental health is mixed, and further research is needed.

#### Predictive validity of the occupational JSI: register data results

**Fig. 2:**
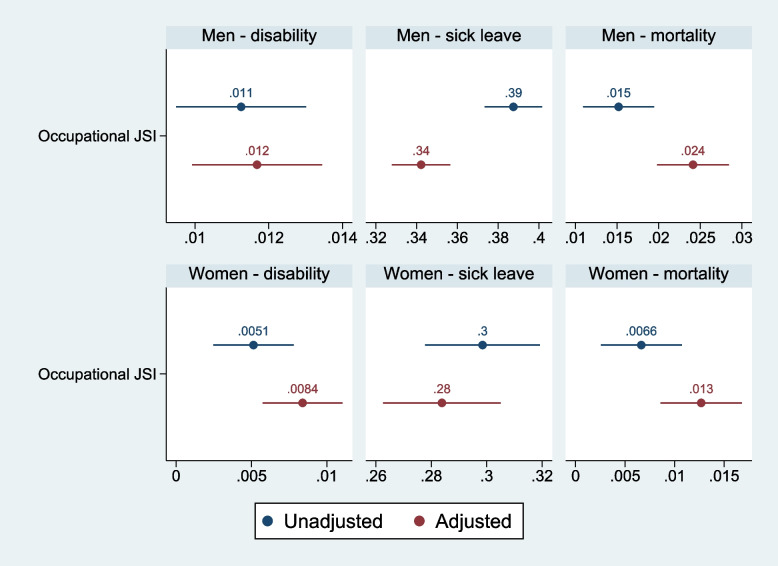
Linear probability model^*^ of receipt of disability (2008–2017), mortality (2008–2017), and long-term sick leave periods (2008–2015) as dependent variables. Register data. ^*^Results unadjusted and adjusted for the level of education and age. Men *N* = 819,232. Women *N* = 770,303

Figure 2 shows the results of linear probability models where the occupational-level JSI is regressed on disability, long-term sick leave, and mortality based on register data, including results for unadjusted and adjusted levels of education and age.

The results showed that both the unadjusted and adjusted occupational-based JSI significantly predicted the likelihood of disability, long-term sick leave, and mortality for both men and women. There were no significant differences between the unadjusted and adjusted occupational-level JSI estimates for both genders when assessing the predictive validity of the JSI based on register data, indicating that the occupation-based JSI showed a reproducible likelihood for disability, sick leave, and mortality.

### Summary and discussion

In this paper, we investigated the reliability and validity of our constructed psychosocial JEM, i.e., the JSI. These assessments involved comparisons of individual job strain with occupational job strain, and of their respective psychosocial dimensions and components, as well as an appraisal of the reliability and criterion validity of the occupational JSI itself. Measured by kappa, agreement between individual-based and occupation-based psychosocial exposures was poor to fair. However, the internal consistency of the two dimensions that make up occupation-based job strain, job demand, and job control was clearly acceptable. According to the factor analysis, the construct validity of the JEM was also fully acceptable. As for concurrent validity, assessed by the survey data, individual- and occupation-based job strain were significantly associated with anxiety and depression for men. For women, the significant associations between job strain and anxiety and between job strain and depression were observed only for individual-based job strain but not for occupation-based job strain. With respect to predictive validity, occupation-based job strain was significantly related to all three health outcomes (disability, sick leave, and mortality) in the register data for both genders.

The results pertaining to the reliability of the JSI were somewhat mixed. The measures that compared individual exposures and occupation-based exposures (kappa, sensitivity, and specificity) tended to be poor, although they varied. On the other hand, the measure of consistency of the two dimensions of job strain performed well. The interpretation of the results related to agreement, sensitivity, and specificity is not straightforward since no gold standard exists. In other words, since individual psychosocial estimates cannot be perceived as the gold standard, poor agreement is subject to several interpretations. This may imply that occupation-based results are far from the mark, but it may equally be that they are close to the mark due to systematic bias in the individual estimates. Hence, due to these interpretive challenges, we would argue that poor agreement and occasionally low sensitivity and specificity do not provide evidence implying that our measures of occupation-based job strain, or job demand or job control were unreliable [36, 37].

Our positive results regarding the predictive validity of the JSI corresponded well with previous studies examining the validity of the JEM in other countries, such as the French psychosocial JEM [11] and the Finnish psychosocial JEM [12]. Since the ultimate purpose of this paper was to construct a validated measure of occupation-based psychosocial work environments for use in register data, we find this specific result rather assuring. We are inclined to put more trust in this finding than in the findings emanating from the analysis of the survey data, which were more mixed. Evidence pertaining to future outcomes (the predictive aspect) is generally considered more robust than evidence related to associations established in cross-sectional data (the concurrent aspect) because of the common variance problem [36]. See also the discussion of limitations below.

Somewhat surprisingly, the occupation-based job strain indicated an elevated risk of anxiety and depression among men but not among women. This does not agree with earlier results showing that higher levels of anxiety and depression were typically reported for women rather than for men [38, 39]. There are two plausible explanations for this gender difference. First, women may be more familiar with working conditions in high-stress and female-dominated occupations than men, such as teachers, social workers, and nurses [40]. Hence, women may tend to underreport their exposure at work compared with men, while mental health outcomes are reported to be higher for women than for men (see descriptive statistic results, page 9–10). There is also evidence that male nurses report more work-related disturbances than female nurses [41], and men working in traditional female jobs may perceive a higher level of social stress than women due to their internalization of the masculinity role [42]. Second, there is evidence of gender differences in job satisfaction, i.e., that men have more difficulties in achieving job satisfaction and are also more willing to express frustration with working conditions than women [43]. Thus, our results suggest that an occupation-based JSI may enhance the ability to identify gender differences in the effect of job strain on health outcomes better than an individual JSI.

Although our results support the idea that a JEM is a reproducible and efficient method for examining work-related health risks in epidemiological studies, some limitations should be considered. The JEMs were converted from individual exposure measurements, which may lead to errors in the JEM assignments due to the imprecise information of exposure for each job and other errors in job coding and duration for individuals [44]. Furthermore, one may argue that JEM is only helpful when job demands within an occupation are comparable, and because JEM assigns the same exposure estimates to all workers with identical job titles, which may affect inter-individual variability, especially in cases where workers have specific tasks [3], or in the case of digitalization of jobs. Another caveat using the JEM developed by the survey data is the risk of differential misclassification. The risk of misclassification is likely to increase when exposure and health outcomes are assessed simultaneously. The individual characteristics of the workers may additionally contribute to the error in self-reported questionnaires in the sense that workers who constantly “complain about everything” may overreport their working exposures and the situation of their health, while another group who “complain about nothing” may underreport their occupational environment and health [36]. This approach may also increase subjective bias and the threats of false positive results, as it reflects the individual perception of the work exposure and health outcomes [45] in cases where workers with health problems tend to report a higher degree of psychosocial exposures than healthy workers. Hence, despite the fact that JEM may provide more objective measures for occupational exposure than self-reported information, this method cannot be seen as a gold standard measure for examining job exposure at work [36, 37]. As discussed above, neither method can. Our study only constructed a JEM based on Norwegian data; thus, it is only appropriate for generalization within Norway and countries that share the same conditions as Norway. To achieve a better applicable JEM, the idea of constructing an international-level JEM (Job Exposure Matrix International-JEMINI) should be further developed [46].

We used the same approach as Hanvold et al. [4] and Solovieva [12] when defining the exposed versus non-exposed as basis for constructing the JEM. Using the median as a cut-off point may, however, be somewhat arbitrary. Thus, in further developments of the JEM one should experiment with different cut-off points to identify possible thresholds for increased health risks.

The JSI could have been constructed by dividing demands by decision latitude which, in contrast to our chosen approach, would have given distributions approaching second degree functions (hyperboles). The advantage of such an approach is the avoidance of defining subjects in extreme "active" and "passive" groups. With our chosen approach there is a risk of labeling subjects as exposed to job strain, who have extremely high demands and rather high control as well and in the other end those who have low demands and extremely low control. However, we have no reason to believe this being an issue of any significance for the results presented in this paper. Dividing demands by decision latitude would exclude more of such problem cases. In further development of the JSI, dividing demands by decision latitude should also be tested.

The Norwegian labor force remains gender- and class-segregated [47]. Our study also indicated that men and women have distinct patterns of psychosocial job exposure that may stem from certain occupations, such as nursing. Although current scholarship has documented evidence of the relationship between job strain, occupational class, and gender [48], few studies have used JEMs. The question of how the risks for different health outcomes are explained by job exposures differentiated by gender and occupational class remains unanswered in our study. Hence, one recommendation is that future research on occupational epidemiology should consider both gender and occupational class when investigating the risk of occupational exposure to health.

## Conclusion

In this study, we assessed certain central aspects of reliability and validity pertaining to an occupation-based JSI, capturing adverse combinations of job demands and job control. The main conclusion of the examination of its statistical properties is that it can be used as an indicator of psychosocial job exposure in Norwegian register data where individual information on psychosocial work environments is missing.

## Data Availability

The data that support the findings of this study are available from Statistics Norway, but restrictions apply to the availability of these data, which were used under license for the current study, and so are not publicly available. Data are however available from the authors upon reasonable request and with permission of the Norwegian Data Protection Official for the Research (NSD) and the Norwegian Data Protection Authority (Datatilsynet).

## Abbreviations

JSI: Job strain index
JEM: Job exposure matrix
N: Number of observations
STYRK-98: Standard for occupational classification used from 1998
STYRK-08: Standard for occupational classification used from 2008
QPS_Nordic_: The General nordic questionnaire
CFA: Confirmative factor analysis
RMSEA: Root mean square error of approximation
CFI: Comparative fit index
TLI: Tucker-Lewis index
SRMR: Standardized root mean square residual
p: Probability
X^2^: Chi-square

## References

[1] Van Der Wel KA, Östergren O, Lundberg O, Korhonen K, Martikainen P, Andersen AN, Urhoj SK (2019). A gold mine, but still no Klondike: Nordic register data in health inequalities research. Scand J Public Health.

[2] Flachs EM, Petersen SEB, Kolstad HA, Schlünssen V, Svendsen SW, Hansen J, Budtz-Jørgensen E, Andersen JH, Madsen IEH, Bonde JPE (2019). Cohort Profile: DOC*X: a nationwide Danish occupational cohort with eXposure data - an open research resource. Int J Epidemiol.

[3] Peters S (2020). Although a valuable method in occupational epidemiology, job-exposure -matrices are no magic fix. Scand J Work Environ Health.

[4] Hanvold TN, Sterud T, Kristensen P, Mehlum IS (2019). Mechanical and psychosocial work exposures: the construction and evaluation of a gender-specific job exposure matrix (JEM). Scand J Work Environ Health.

[5] Kauppinen TP, Mutanen PO, Seitsamo JT (1992). Magnitude of misclassification bias when using a job-exposure matrix. Scand J Work Environ Health.

[6] Karasek RA (1979). Job Demands, Job Decision Latitude, and Mental Strain: Implications for Job Redesign. Adm Sci Q.

[7] Pelfrene E, Vlerick P, Mak RP, de Smet P, Kornitzer M, De Backer G (2001). Scale reliability and validity of the Karasek 'Job Demand-Control-Support' model in the Belstress study. Work Stress.

[8] Sanne B, Torp S, Mykletun A, Dahl AA (2005). The Swedish Demand-Control-Support Questionnaire (DCSQ): factor structure, item analyses, and internal consistency in a large population. Scand J Public Health.

[9] Courvoisier DS, Perneger TV (2010). Validation of alternative formulations of job strain. J Occup Health.

[10] Milner A, Niedhammer I, Chastang JF, Spittal MJ, LaMontagne AD (2016). Validity of a Job-Exposure Matrix for Psychosocial Job Stressors: Results from the Household Income and Labour Dynamics in Australia Survey. PLoS ONE.

[11] Niedhammer I, Chastang JF, Levy D, David S, Degioanni S, Theorell T (2008). Study of the validity of a job-exposure matrix for psychosocial work factors: results from the national French SUMER survey. Int Arch Occup Environ Health.

[12] Solovieva S, Pensola T, Kausto J, Shiri R, Heliövaara M, Burdorf A, Husgafvel-Pursiainen K, Viikari-Juntura E (2014). Evaluation of the validity of job exposure matrix for psychosocial factors at work. PLoS ONE.

[13] Niedhammer I, Milner A, LaMontagne AD, Chastang JF (2018). Study of the validity of a job–exposure matrix for the job strain model factors: an update and a study of changes over time. Int Arch Occup Environ Health.

[14] Hartikainen E, Solovieva S, Viikari-Juntura E, Leinonen T (2022). Associations of employment sector and occupational exposures with full and part-time sickness absence: random and fixed effects analyses on panel data. Scand J Work Environ Health.

[15] Samuelsson Å, Ropponen A, Alexanderson K, Svedberg P (2013). Psychosocial working conditions, occupational groups, and risk of disability pension due to mental diagnoses: a cohort study of 43,000 Swedish twins. Scand J Work Environ Health.

[16] Niedhammer I, Bertrais S, Witt K (2021). Psychosocial work exposures and health outcomes: a meta-review of 72 literature reviews with meta-analysis. Scand J Work Environ Health.

[17] Bonde JP (2008). Psychosocial factors at work and risk of depression: a systematic review of the epidemiological evidence. Occup Environ Med.

[18] Stansfeld S, Candy B (2006). Psychosocial work environment and mental health–a meta-analytic review. Scand J Work Environ Health.

[19] Madsen IEH, Nyberg ST, Magnusson Hanson LL, Ferrie JE, Ahola K, Alfredsson L, Batty GD, Bjorner JB, Borritz M, Burr H, Chastang JF, de Graaf R, Dragano N, Hamer M, Jokela M, Knutsson A, Koskenvuo M, Koskinen A, Leineweber C, Niedhammer I, Nielsen ML, Nordin M, Oksanen T, Pejtersen JH, Pentti J, Plaisier I, Salo P, Singh-Manoux A, Suominen S, Ten Have M, Theorell T, Toppinen-Tanner S, Vahtera J, Väänänen A, Westerholm PJM, Westerlund H, Fransson EI, Heikkilä K, Virtanen M, Rugulies R, Kivimäki M; IPD-Work Consortium. Job strain as a risk factor for clinical depression: systematic review and meta-analysis with additional individual participant data. Psychol Med. 2017;47(8):1342–56. 10.1017/S003329171600355X. Epub 2017 Jan 26. 10.1017/S003329171600355X PMC5471831 28122650

[20] Theorell T, Hammarström A, Aronsson G, TräskmanBendz L, Grape T, Hogstedt C, Marteinsdottir I, Skoog I, Hall C (2015). A systematic review including meta-analysis of work environment and depressive symptoms. BMC Public Health.

[21] Linton SJ, Kecklund G, Franklin KA, Leissner LC, Sivertsen B, Lindberg E, Svensson AC, Hansson SO, Sundin Ö, Hetta J, Björkelund C, Hall C (2015). The effect of the work environment on future sleep disturbances: a systematic review. Sleep Med Rev.

[22] Yang B, Wang Y, Cui F, Huang T, Sheng P, Shi T, Huang C, Lan Y, Huang YN (2018). Association between insomnia and job stress: a meta-analysis. Sleep Breath.

[23] Wieclaw J, Agerbo E, Mortensen PB, Burr H, Tuchsen F, Bonde JP (2008). Psychosocial working conditions and the risk of depression and anxiety disorders in the Danish workforce. BMC Public Health.

[24] Norberg J, Alexanderson K, Framke E, Rugulies R, Farrants K (2020). Job demands and control and sickness absence, disability pension and unemployment among 2,194,692 individuals in Sweden. Scand J Public Health.

[25] Statistics Norway. Standard Classification of Occupations. 1998. https://www.ssb.no/a/publikasjoner/pdf/nos_c521/nos_c521.pdf.

[26] Statistics Norway. Standard Classification of Occupations (STYRK-08). 2011. https://www.ssb.no/a/publikasjoner/pdf/notat_201117/notat_201117.pdf?msclkid=0bebc964a9d911ec850a2dd2036eefb7RK-08) (ssb.no).

[27] Skogstad A, Knardahl S, Lindström K, Elo A, Dallner M, Gamberale F, Hottinen V, Ørhede E. Brukerveileding QPSNordic: Generelt spørreskjema for psykologiske og sosiale faktorer i arbeid. STAMI-rapport Årg. 1, nr. 2 (2001). https://www.qps-nordic.org/no/doc/Brukerveiledning_qpsnordic.pdf-man-N-F (qps-nordic.org).

[28] Landsbergis P, Theorell T, Schwartz J, Greiner BA, Krause N (2000). Measurement of psychosocial workplace exposure variables. Occup Med.

[29] Theorell T, Perski A, Akerstedt T, Sigala F, Ahlberg-Hultén G, Svensson J, Eneroth P (1988). Changes in job strain in relation to changes in physiological state. A longitudinal study Scand J Work Environ Health.

[30] Chungkham HS, Ingre M, Karasek R, Westerlund H, Theorell T (2013). Factor structure and longitudinal measurement invariance of the demand control support model: an evidence from the Swedish Longitudinal Occupational Survey of Health (SLOSH). PLoS ONE.

[31] Cohen J (1968). Weighted kappa: nominal scale agreement with provision for scaled disagreement or partial credit. Psychol Bull.

[32] Karasek R, Brisson C, Kawakami N, Houtman I, Bongers P, Amick B (1998). The Job Content Questionnaire (JCQ): an instrument for internationally comparative assessments of psychosocial job characteristics. J Occup Health Psychol.

[33] Mauss D, Herr RM, Theorell T, Angerer P, Li J (2018). Validating the Demand Control Support Questionnaire among white-collar employees in Switzerland and the United States. J Occup Med Toxicol.

[34] Browne MW, Cudeck R. Alternative ways of assessing model fit. In: Bollen K and Long J (eds) Testing structural equation models. London: Sage, 1993.

[35] Hu L, Bentler P (1999). Cutoff criteria for fit indexes in covariance structure analysis: conventional criteria versus new alternatives. Struct Equ Modeling.

[36] Theorell T, Hasselhorn HM (2005). On cross-sectional questionnaire studies of relationships between psychosocial conditions at work and health–are they reliable?. Int Arch Occup Environ Health.

[37] Solovieva S, Pehkonen I, Pensola T, Haukka E, Kausto J, Leivategija T, Shiri R, Heliövaara M, Burdorf A, Husgafvel‐Pursiainen K, Viikari-Juntura E. Development of physical and psychosocial job exposure matrices. Finish institute of occupational Health. Helsinki 2014. https://www.julkari.fi/bitstream/handle/10024/135076/Development%20of%20physical%20and%20psychosocial%20job%20exposure%20matrices.pdf?sequence=1&isAllowed=yf (julkari.fi).

[38] Jenkins R, Lewis G, Bebbington P, Brugha T, Farrell M, Gill B, Meltzer H (1997). The National Psychiatric Morbidity surveys of Great Britain–initial findings from the household survey. Psychol Med.

[39] Marcus SM, Young EA, Kerber KB, Kornstein S, Farabaugh AH, Mitchell J, Wisniewski SR, Balasubramani GK, Trivedi MH, Rush AJ (2005). Gender differences in depression: findings from the STAR*D study. J Affect Disord.

[40] Wieclaw J, Agerbo E, Mortensen PB, Bonde JP (2006). Risk of affective and stress related disorders among employees in human service professions. Occup Environ Med.

[41] Evans O, Steptoe A (2002). The contribution of gender-role orientation, work factors and home stressors to psychological well-being and sickness absence in male- and female-dominated occupational groups. Soc Sci Med.

[42] Sobiraj S, Rigotti T, Weseler D, Mohr G (2015). Masculinity ideology and psychological strain: Considering men’s social stressors in female-dominated occupations. Psychol Men Masc.

[43] Hodson R (1989). Gender Differences in Job Satisfaction: Why Aren’t Women More Dissatisfied?. The Sociological Quarterly.

[44] Greenland S, Fischer HJ, Kheifets L (2016). Methods to Explore Uncertainty and Bias Introduced by Job Exposure Matrices. Risk Anal.

[45] Blair A, Stewart P, Lubin JH, Forastiere F (2007). Methodological issues regarding confounding and exposure misclassification in epidemiological studies of occupational exposures. Am J Ind Med.

[46] Descatha A, Evanoff BA, Andersen JH, Fadel M, Ngabirano L, Leclerc A, Dale AM, Roquelaure Y (2019). JEMINI (Job Exposure Matrix InterNatIonal) Initiative: a Utopian Possibility for Helping Occupational Exposure Assessment All Around the World?. J Occup Environ Med.

[47] Hall EM (1989). Gender, work control, and stress: a theoretical discussion and an empirical test. Int J Health Serv.

[48] Kawakami N, Haratani T, Kobayashi F, Ishizaki M, Hayashi T, Fujita O, Aizawa Y, Miyazaki S, Hiro H, Masumoto T, Hashimoto S, Araki S (2004). Occupational class and exposure to job stressors among employed men and women in Japan. J Epidemiol.

